# Hands-on false memories: a combined study with distributional semantics and mouse-tracking

**DOI:** 10.1007/s00426-022-01710-x

**Published:** 2022-07-18

**Authors:** Daniele Gatti, Marco Marelli, Giuliana Mazzoni, Tomaso Vecchi, Luca Rinaldi

**Affiliations:** 1 Department of Brain and Behavioral Sciences, University of Pavia, Piazza Botta 6, 27100 Pavia, Italy; 2 Department of Psychology, University of Milano-Bicocca, Milan, Italy; 3 NeuroMI, Milan Center for Neuroscience, Milan, Italy; 4 Faculty of Medicine and Psychology, University La Sapienza, Rome, Italy; 5 School of Life Sciences, University of Hull, Hull, UK; 6 Cognitive Psychology Unit, IRCCS Mondino Foundation, Pavia, Italy

## Abstract

Although mouse-tracking has been seen as a real-time window into different aspects of human decision-making processes, currently little is known about how the decision process unfolds in veridical and false memory retrieval. Here, we directly investigated decision-making processes by predicting participants’ performance in a mouse-tracking version of a typical Deese–Roediger–McDermott (DRM) task through distributional semantic models, a usage-based approach to meaning. Participants were required to study lists of associated words and then to perform a recognition task with the mouse. Results showed that mouse trajectories were extensively affected by the semantic similarity between the words presented in the recognition phase and the ones previously studied. In particular, the higher the semantic similarity, the larger the conflict driving the choice and the higher the irregularity in the trajectory when correctly rejecting new words (i.e., the false memory items). Conversely, on the temporal evolution of the decision, our results showed that semantic similarity affects more complex temporal measures indexing the online decision processes subserving task performance. Together, these findings demonstrate that semantic similarity can affect human behavior at the level of motor control, testifying its influence on online decision-making processes. More generally, our findings complement previous seminal theories on false memory and provide insights into the impact of the semantic memory structure on different decision-making components.

**Supplementary Information:**

The online version contains supplementary material available at 10.1007/s00426-022-01710-x.

## Introduction

Human memory is not simply a precise tape recorder, but a system that encodes and ultimately represents knowledge through a process of active reconstruction rather than passive reproduction (Schacter, 2001; Vecchi & Gatti, 2020). This was first documented in Bartlett’s pioneering studies on memory (1932) and supported by a large body of subsequent research, in which it was demonstrated that participants would forget the precise features of the stimuli they have memorized, in favor of an extraction of the gist of the information (for a review: Brainerd et al., 2008a; Chang & Brainerd, 2021). That is, humans would use their semantic memory to encode, store and retrieve information, adapting new information to what has been previously memorized, with systematic errors that may occur during these phases (Brewer & Treyens, 1981; Sulin & Dooling, 1974).

The origins of inaccurate or false memories have therefore gradually become a matter of great scientific interest. Different experimental paradigms have been developed to account for false memories’ formation, with the Deese–Roediger–McDermott (DRM; Deese, 1959; Roediger & McDermott, 1995) task being one of the most widely used method in the verbal domain. In this task, participants are typically first presented once with several lists of words that have to be memorized (within each list, the words are related to a non-shown target word, named critical lure; e.g., word list: *door*, *glass*, *pan*, *shade*, *ledge*, etc.—critical lure: *window*) and then, after a brief distracting task, they are asked to perform a recognition task in which they have to indicate whether a given word was part of the memorized lists or not. Interestingly, during this latter phase, participants tend to erroneously report as “old” the critical lures, (i.e., they recognize them as if they were part of the memorized lists, although these words were never presented during the encoding phase; for a review, see: Gallo, 2010).

To explain participants’ performance in the DRM task, two main theories have been proposed: activation-monitoring framework (Gallo & Roediger, 2002; Roediger et al., 2001) and fuzzy-trace theory (Brainerd & Reyna, 2002; Reyna & Brainerd, 1995). According to activation-monitoring framework, the critical lure would be hyperactivated by the presentation of the studied words related to it, thus leading to high levels of false recognition (Roediger et al., 2001). Conversely, according to fuzzy-trace theory, while studying the words participants would encode a memory trace—called gist trace—linked to the semantic content of each list, which would be responsible for the production of the false recognitions (Brainerd & Reyna, 2002; Reyna & Brainerd, 1995).

Consistent with these perspectives, previous studies have successfully predicted false memories occurrence on associative (i.e., associative relationships reflect word use, such as “spider-web”) and semantic (i.e., semantic relationships reflect overlap of conceptual features between words, such as “horse-pony”) bases (Brainerd et al., 2008b; Roediger et al., 2001). In particular, seminal studies have shown that the association strength between the words that compose each list and the critical lure (i.e., the backward associative strength, BAS) is a key factor in determining false memories (Roediger et al., 2001) and that multiple semantic sub-components underlie false memories (Cann et al., 2011). The debate around the associative vs. semantic nature of false memories in the DRM is igniting fervent discussions (for recent evidence see: Brainerd et al., 2020), as generally these two processes are considered to be independent (Ferrand & New, 2003; Hutchison, 2003). However, recent studies have shown that indexes extracted from natural language use are successful in predicting both associative and semantic effects in priming tasks (Günther, et al., 2016; Jones et al., 2006). Such evidence, indicating that the same process (i.e., predicting a target word from the linguistic context in which it typically appears) can explain both associative and semantic processing, suggests in turn that these two processes are likely interdependent in natural settings and may converge into (partially) overlapping structural representations of human memory. That is, the dissociation between associative and semantic processes would be possible on an experimental level by forcing participants to rely on a specific component given certain task demands (e.g., defining a concept or guessing at associates necessarily forces participants to rely on different components of human memory; for a discussion see, Maki & Buchanan, 2008); yet, in natural contexts it is almost impossible to isolate such components and thus to find words that are purely semantically or associatively related (Jones et al., 2006).

However, currently little is known about how the decision process unfolds when participants accept or reject words in the DRM task. That is, accuracy and reaction times—the classic dependent variables used in memory research—although informative of some explicit and implicit cognitive components are associated with the final state of the decision process, and hence cannot provide a direct measure of how this process unfolds or cannot directly quantify potential conflicts in the response (Freeman, 2018; Stillman et al., 2018). Alternative methods, such as drift-diffusion models (e.g., Krajbich & Rangel, 2011; Ratcliff, 1978; for evidence on memory tasks see: Osth et al., 2020), can be used to isolate certain decision components. Specifically, based on reaction times distributions, drift-diffusion models can be for example used to estimate (i) how much the responses of a given participant in a certain condition are relatively conservative/biased, (ii) the degree of perceptual sensitivity or task difficulty, or (iii) the duration of non-decisional components within the decision process (e.g., for further details see: Voss et al., 2004). The adoption of these models has consequently allowed to clarify several decision components within a large number of psychological domains, including human memory (e.g., Osth et al., 2020). However, one limit of drift diffusion models is that, in order to estimate the parameters with high quality, such models require a high number of observations within each condition (Voss et al., 2004). This, in turn, prevents the adoption of continuous predictors at the single stimulus-level (like the semantic predictor employed in the present study).

For these reasons, here we opted for using mouse tracking, as a particularly reliable method able to isolate the dynamics of response conflict and indecision, as well as the evolution of the choice (Freeman, 2018), allowing also for the computation of trial-level estimates. Accordingly, it has also been shown that mouse-tracking measures outperform reaction times in predicting participant’s performance in decisions involving risk (Stillman et al., 2020). In recent years, mouse-tracking has been indeed successfully used to investigate how participants’ decision unfolds across several cognitive domains such as language (Lins et al., 2019; Spivey et al., 2005), social cognition (Freeman et al., 2011, 2016), recognition memory (Papesh et al., 2012, 2019), and also to detect faking-good behavior when responding to personality questionnaires (Mazza et al., 2020). Recently we employed a mouse-tracking paradigm to investigate real-time decisions during semantic processing, by predicting participants’ performance through distributional semantics (Gatti et al., 2021b). Specifically, in this study participants were shown word pairs and were required to perform a two-alternative forced-choice task selecting either the more abstract or the more concrete word, with the response selection that was achieved by moving the computer mouse. Results showed that mouse trajectories reflected the response conflict and its temporal evolution, with a larger deviation for increasing word semantic relatedness, thus supporting the validity of mouse-tracking as a method to detect deep and implicit decision-making features subserving semantic memory (Gatti et al., 2021b).

In mouse-tracking paradigms, participants are required to make decisions by moving their mouse from a starting position (typically placed in the middle-bottom part of the screen) to one of the two options presented (typically placed in the two upper corners of the screen). It is assumed that motor outputs (i.e., hand movements) are executed in parallel with the decision that participants are required to make (Freeman et al., 2010), thus allowing for the quantification of the conflict of the choice and its evolution, which cannot be directly assessed using only reaction times (Stillman et al., 2018). Through mouse-tracking packages (e.g., Kieslich et al., 2019) it is indeed possible to extract several dependent variables that are informative about decision-making processes. Generally, decision conflict is quantified by computing the maximum deviation from the direct path (MD; i.e., the furthest point on the actual trajectory from the idealized straight trajectory between the starting point and the selected stimulus), while the decision evolution is quantified by computing the sample entropy, which measures the irregularity and unpredictability degree of the trajectory (for a complete discussion on other possible indexes see, Freeman & Ambady, 2010; Stillman et al., 2018). For both measures the higher the value, the higher the conflict and the level of indecision. Additionally, mouse-tracking paradigms allow for more refined decision time indexes, such as the computation of the time at which the trajectory reaches the maximum distance (the time at which the decision takes place). Participants are not subjectively aware that their manual trajectories are deviating as a function of task conditions, and thus these indexes can be considered as quantifying implicit and likely automatic measures. Consistent with this, within the recognition domain, seminal evidence has shown that mouse-tracking measures correlate with confidence judgements, with judgements with higher confidence showing more linear response trajectories (Papesh et al., 2012). However, it should be noted that, compared with classic confidence judgements, mouse-tracking paradigms provide continuous (and not ordinal) dependent variables, thus allowing for more straightforward analyses. Additionally, previous evidence on lexical decision tasks has also shown that entropy quantifies indecision and thus provides additional information on decisional processes compared with other decisional measures (Calcagnì et al., 2017). Other direct evidence comes from seminal studies investigating the conflict in lexical decision judgements and showing that during the categorization of the atypical exemplar “whale” as “mammal” (vs. the tempting yet incorrect option, “fish”), participants’ movements were more attracted from the “fish” option than when the target was a typical mammal (Dale et al., 2007).

Here, building upon this evidence, we take advantage of mouse-tracking paradigms to explore the decisional stages subserving recognition memory in the DRM task. Specifically, we applied an already established method to compute the semantic similarity between the new words and the studied words (for a complete discussion see: Gatti et al., 2021a, 2021b, 2021c), by employing indexes extracted from distributional semantic models (DSMs). DSMs induce words meanings from large databases of natural language data, representing them as high-dimensional numerical vectors: these models are indeed thought to well capture the structure of semantic memory (Günther et al., 2019; Jones et al., 2015). In particular, here we used word-embeddings that are based on a predictive component: these DSMs induce word vectors using a neural network architecture with one hidden layer, which is optimized to match a target word (Baroni et al., 2014; Mikolov et al., 2013). Briefly, these models are trained on large collections of texts that document natural language use. Nodes in the input and output layers represent words, and a neural network learns to predict a target word on the basis of the lexical contexts in which it appears (i.e., the words it co-occurs within the text), incrementally updating a set of weights by minimizing the difference between model predictions and observed data at each learning event (i.e., every occurrence of the target word). The estimated sets of weights will eventually capture word meanings. These distributed representations, or vectors, can be quantitatively compared by measuring their distance in a multidimensional space, which in turn is thought to capture the semantic similarity between words (Günther et al., 2019): similar words will occur in similar contexts, ending up being associated with vectors that are geometrically closer. Importantly, word embeddings have been shown to be high-performing across a wide range of semantic tasks (for a review on the recent prediction-based class of models, see e.g., Baroni et al., 2014). Moreover, they are equivalent to psychologically grounded associative learning models (Günther et al., 2019; Mandera et al., 2017).

While previous studies predicted participants’ performance in the DRM task mainly by adopting human-based measures (e.g., backward associative strength—BAS; Roediger et al., 2001), here we thus employed a measure not computed on human ratings, but rather automatically extracted from natural language. It should be noted that BAS and DSMs-based metrics in the DRM task have been shown to be correlated (i.e., *r* = 0.50, see Gatti et al., 2022 for an in-depth discussion regarding such a relationship). However, the adoption of an independent-source measure such as data from DSMs may be preferable: that is, predicting human performance using data from association norms in a task that is necessarily tapping on the cognitive processes generating such norms (i.e., as most DRMs are explicitly constructed from free-association norms) may lead to explanatory circularity (Westbury, 2016). In line with this view, here we aimed to predict participants’ behavior in the DRM task starting from independent models that replicate the structure of semantic memory by applying a psychologically-plausible learning model to environmental regularities (i.e., word co-occurrences) (Günther et al., 2019).

Participants were asked to study several lists of words from a classic DRM task and then, in the recognition phase, they were asked to indicate using their mouse if the words showed were “old” (i.e., presented in the encoding phase) or “new” (i.e., not previously presented). The spatial and temporal measures extracted from mouse movements were then predicted using a semantic index extracted from a DSM. This method allowed us to investigate whether the decision process differs depending on the position of the new (and studied) words in the semantic space (i.e., whether words in the recognition phase are more semantically similar or not to the studies words).

## Methods

### Participants

Seventy-one students participated in the study (28 males, *M* age = 24.4 years, SD = 3.63, age range = 19–35). All participants were native Italian speakers, had normal or corrected to normal vision and were naïve to the purpose of the study. Informed consent was obtained from all participants before the experiment. The protocol was approved by the psychological ethical committee of the University of Pavia and participants were treated in accordance with the Declaration of Helsinki.

### Stimuli

We used the DRM task (Deese, 1959; Roediger & McDermott, 1995), a typical false memories paradigm. Participants were first instructed to memorize several lists of words and then to perform a recognition task. The words that composed each list were associatively related to a non-shown word (called critical lure).

For the encoding phase, we selected 12 lists of words out of 24 from the normative data for the Italian DRM test (Iacullo & Marucci, 2016). Each list was originally composed of 15 words: we selected the first 12 words (144 words in total), while 2 of the 3 remaining words were used as weakly related lures.

The recognition phase was composed of 96 words, 48 of which had been presented in the previous phase (i.e., studied words) and 48 of which had not been previously presented (i.e., new words). The 48 studied words presented in this experimental phase were those in serial positions 1, 4, 7 and 10 in the studied lists. Of the 48 new words, 12 were the critical lures from the studied lists (i.e., the non-shown words mostly associated with the words composing each list), 24 were weakly related lures and 12 were unrelated words. The weakly related lures were 2 of the 3 words of the studied lists that were not presented in the list, specifically those in position 13 and 14. The unrelated words were chosen randomly among the words of the excluded lists; this criterion was established arbitrarily (for a similar method, see: Gatti et al., 2022).

### Procedure

Participants were tested online using Psychopy (Peirce, 2007, 2009; Peirce & MacAskill, 2018; Peirce et al., 2019) through the online platform Pavlovia (https://pavlovia.org/). Only participants performing the task with an external mouse were included (i.e., those with a trackpad were excluded a priori).

During the first part of the task, participants had to memorize a series of words (i.e., they were required to study 12 lists of words without interruptions). Participants were shown the 12 words that composed each of the 12 lists in descending forward associative strength (FAS; i.e., the association strength from the critical lure to the word that composes the list). The order by which the lists were presented was random, while the order of the words within each list was fixed according to the FAS (see: Iacullo & Marucci, 2016). Each trial started with a central fixation cross (presented for 500 ms) followed by a word (presented for 1500 ms) and a blank screen (presented for 300 ms), then the script moved automatically to the next fixation cross.

At the end of the encoding phase, participants were required to perform an attentional task (i.e., a modified version of the go-no-go) as a distracting task for 2 min.

**Fig. 1:**
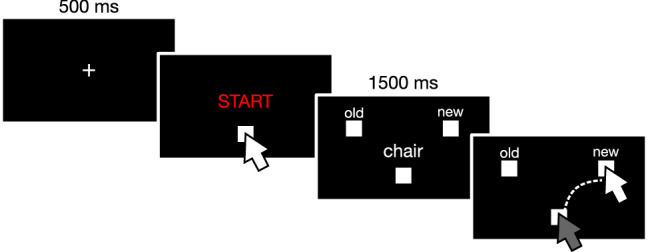
Recognition task. Participants were asked to make old/new judgments using their mouse. After clicking on the START button, they were shown a word and were required to make the memory judgement (i.e., indicate whether the target word was previously presented or not during the encoding phase)

Then participants were asked to perform the recognition phase. Participants were instructed to make old/new judgments using their mouse: participants had to first click on a square presented below the “START” label by pressing the left button of the mouse, and next to move to the selected option (old or new), again pressing the left button to make their decision. Each trial began with a fixation cross presented at the center of the screen (500 ms), then “START” appeared at the bottom-center of the screen (Arial red font, white button to be pressed located at: *x* = 0, *y* = − 0.35); after the participants clicked on it, the word to be recognized appeared just above the starting button (shown for 1500 ms; Arial font, *x* = 0, *y* = 0), while “old” and “new” buttons appeared in the upper right and upper left of the screen (old/new buttons always had the same location within the same participant, locations were counterbalanced across participants, Arial font for the old/new; buttons located at: *x* =  ± 0.50, *y* = 0.25; see Fig. 1). Participants were asked to initiate their physical movement as fast as possible.

### Distributional semantic model

Vector representations for the words used in this study were extracted from an Italian DSM obtained via the application of neural networks, and in particular the Continuous Bag of Words method, an approach originally proposed by Mikolov et al. (2013): when using Continuous Bag of Words, the obtained vector dimensions capture the extent to which a target word is reliably predicted by the contexts in which it appears. The model, released by Marelli (2017), was trained on ItWaC, a free Italian text corpus based on web-collected data and consisting of about 1.9 billion tokens (semantic relatedness values are available online via the SNAUT database: http://meshugga.ugent.be/snaut-italian/). The model used is set on the following parameters: *9-word co-occurrence window*, *400-dimension vectors*, negative sampling with *k* = 10, subsampling with *t* = 1e^−5^. This set of parameters defines the learning procedure used to induce word vectors (Mikolov et al., 2013). Co-occurrence window size indicates how large the considered lexical contexts are; in our case, a *9-word window* indicates that we estimated predictions concerning 4 words on the left and 4 words on the right of the target word.

**Fig. 2:**
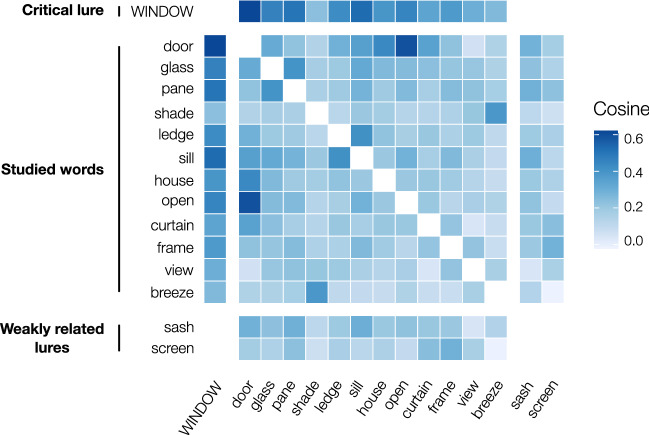
A heatmap matrix of the cosine values among the words composing the list *window* (i.e., critical lure; words list taken from Roediger & McDermott, 1995). Darker blue colors represent higher cosine values (and, hence, account for more semantically related words as predicted by DSMs). Note that in this case we plotted all the words in the list (i.e., including the possible weakly related lures) and that we computed the cosine values through an English DSM (available here: http://meshugga.ugent.be/snaut-english/; see also: Mandera et al., 2017), but that in the study both DRM lists and DSM used to estimate SSim values were Italian

From this semantic space, we extracted vector-based estimates for the word items of the present study.^1^ Specifically, for each word pair it is possible to obtain the cosine of the angle formed by the vectors representing the meanings of these words. The higher the cosine the more semantically similar the words will be. A heatmap matrix of the cosine values among the words composing a reference DRM list is represented in Fig. 2.

### Computation of semantic similarity values

For each new word (12 critical lures, 24 weakly related lures and 12 unrelated words) we computed a semantic similarity index (SSim). That is, SSim was computed as the frequency-weighted average value of the cosines of the angles formed by the vectors representing the meanings of each new word in the recognition phase and each of the 12 words that composed its relative list (for a similar approach see: Gatti et al., 2022; Marelli & Amenta, 2018). For unrelated words, we computed the index randomly matching each word with a list. The formula used was:

$$\mathrm{SSim}(nw)=\frac{\sum_{i=1}^{k}cos(\overrightarrow{nw},\overrightarrow{{sw}_{i}})\times {Fsw}_{i}}{\sum_{i=1}^{k}{Fsw}_{i}}$$

where *nw* is a new word shown during the recognition task, $cos$ refers to the cosine of the angle formed by the vectors representing a new word ($\overrightarrow{nw}$) and each of the *k* studied words ($\overrightarrow{sw}$) composing its list (e.g., for critical lures, we computed all the cosines between the critical lure and each word of its list), while *Fsw*_*i*_ is the frequency of each studied word as extracted from the Italian SUBTLEX (http://crr.ugent.be/subtlex-it/). Following the same rationale, the SSim index for studied words was computed between each studied word presented in the recognition phase and each of the other 11 words that composed its relative list.

### Data analysis

All the analyses were performed using *R*-Studio (RStudio Team, 2015). Data were analyzed through a mixed-effects approach, which incorporates both fixed-effects and random effects (associated with participants and task stimuli) and allows for the specification of predictors at both participants and/or item level. All the generalized linear mixed models (GLMMs) and linear mixed models (LMMs) were run using the *lme4 R* package (Bates et al., 2015). The graphs reported were obtained using the *effects R* package (Fox, 2003; Fox & Weisberg, 2019).

Besides participants’ explicit memory judgements (i.e., “new” responses were scored as 0, whereas “old” responses as 1), our main dependent variables were: maximum deviation from the direct path (MD), sample entropy, initiation reaction times (RTs) and maximum deviation from direct path time (MDtime).

MD is defined as the furthest point on the actual trajectory from the idealized straight trajectory and is thought to quantify the conflict in the choice. Sample entropy is defined as the degree of irregularity and unpredictability in movement across the x-axis and is thought to measure the evolution of the choice (i.e., with higher values indicating higher irregularity). Initiation RT is computed as the time elapsed between the click on the START button and the first-hand movement, while MDtime indexes the time at which the trajectory reaches the MD. These two dependent variables are considered to measure two different time windows of decision-making, with the former (initiation RTs) indicating the time at which the decisional process starts and the latter (MDtime) indicating the time at which a decision is finally achieved (see for a complete discussion regarding mouse-tracking variables: Stillman et al., 2018).

All the mouse-tracking-related dependent variables were computed using the *mousetrap R* package (Kieslich et al., 2019). All trajectories were normalized into 101-time steps and remapped symmetrically to allow for a direct comparison of trajectories which differed in duration and number of data points. Initiation RTs, MDtime and sample entropy were all log-transformed.

First, we aimed to replicate the significant interaction SSim by type of stimuli (new vs. old) on participants’ explicit memory judgements as previously reported (for a complete discussion of this analysis see: Gatti et al., 2022). Participants’ explicit memory judgements were thus analyzed estimating a GLMM with SSim, type of stimuli (new vs. old) and their interaction as predictors; subjects and items were included as random intercepts.

Then, following a similar approach, we included the SSim predictor in the analyses on mouse-tracking dependent variables, estimating four LMMs in which we included in interaction SSim as a continuous predictor, type of stimuli (new vs. old) and participant’s response (new vs. old; i.e., whether the participant judged each word as showed or not) as categorical predictors; subjects and items were included as random intercepts. In case of singularity issues (i.e., for MD, sample entropy and initiation RTs, but not for MDtime), the random model was simplified removing the intercept of the item and then refitted. In particular, in *lme4* syntax, the models tested were:

$$DV \sim SSim*Type*Response+\left(1|Participant\right)+(1|Item)$$

Then, to exclude the impact of overly influential outliers, after having fitted the model, data points were removed on the basis of a threshold of 2.5 *SD* standardized residual errors (model criticism; Baayen, 2008). Results based on the refitted models are reported. Observed power was computed by means of the *simr* R package (Green & MacLeod, 2016), using the function *powerSim* on 1000 simulations and comparing the full model (i.e., the one refitted after model criticism) with the one not including the triple interaction.

## Results

Trials in which overall reaction times (i.e., the time between the initiation of the movement and the selection of one option) were faster than 300 ms or slower than 5000 ms were excluded from the analysis (0.7% of the trials excluded). Aberrant movements (i.e., trials in which the *mousetrap R* package was unable to compute the trajectories) were detected in 6% of additional trials and were discarded. Trials in which MD and sample entropy were ± 3SD from the mean of the participants were removed from the analysis (2% of additional trials excluded).

### The differential effect of SSim on false and veridical recognitions

In the GLMM including recognition explicit responses (i.e., “new” responses were scored as 0, “old” as 1) as a dependent variable, SSim and type of stimuli (studied vs. new words) as predictors, we found significant effects of both variables on recognition (SSim: *z* = 9.84, *p* < 0.001; type of stimuli: *z* = 3.36, *p* < 0.001; *Pseudo-R*^2^ (total) = 0.47). Critically, the interaction SSim by type of stimuli was significant, *z* = − 3.24, *p* = 0.001.

**Fig. 3:**
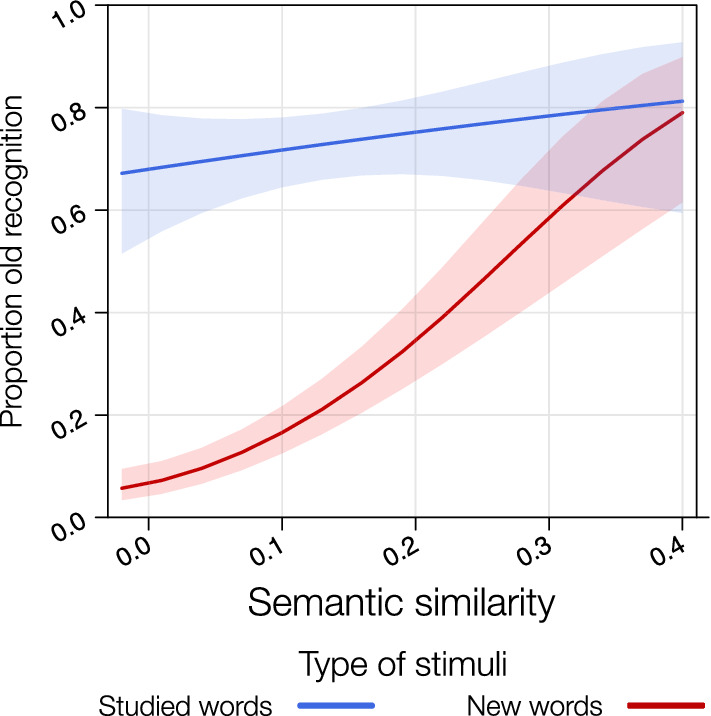
Results from the GLMM including the interaction between SSim and type of stimuli on participants’ proportion of old response (i.e., for new words, the higher the value the higher the occurrence of false memories)

The significant interaction indicates that the effect of SSim was specific for false recognitions, *z* = 6.43, *p* < 0.001, while it was not significant for veridical recognitions, *z* = 0.90, *p* = 0.37 (Fig. 3). In particular, for new words, the higher the SSim value, the higher the occurrence of false memories, in line with previous findings (Gatti et al., 2022).

### Analyses on mouse-tracking dependent variables

#### Descriptive statistics

**Fig. 4:**
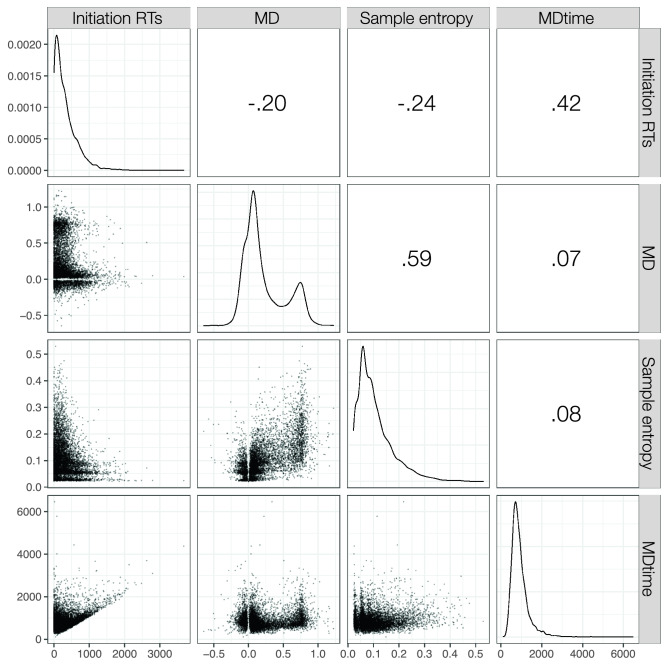
Correlation matrix of the four dependent variables included in the current study. The distributions are reported in the diagonal, the correlations coefficients are reported in the upper triangle, and the scatterplots are reported in the lower triangle. All *p*s < 0.01, 6201 degrees of freedom

Descriptive statistics for the four variables are reported in Table 1. The correlation matrix between the four dependent variables is reported in Fig. 4.^2^ Overall, the correlations ranged from very low, as the one between MD and MDtime (*r* = − 0.06), to high, as the one between MD and sample entropy (*r* = 0.59). Note that the correlations were computed on raw data, hence on a total of 6203 trials.

**Table 1:** Descriptive statistics for the four dependent variables analyzed. The descriptive statistics of the two variables expressing temporal processes (initiation RTs and MDtime) are reported in milliseconds

	Initiation RTs	MD	Sample entropy	MDtime
Mean	330	0.22	0.10	917
SD	197	0.12	0.03	204
Min–max	51–907	− 0.01 to 0.48	0.05–0.90	554–1402

#### Initiation reaction times

In the LMM including Initiation RTs as a dependent variable, SSim, type of stimuli and participant’s response as predictors, we found no significant effect, all *p*s > 0.20. Observed power = 21.60%.

#### Maximum deviation from a direct path

**Fig. 5:**
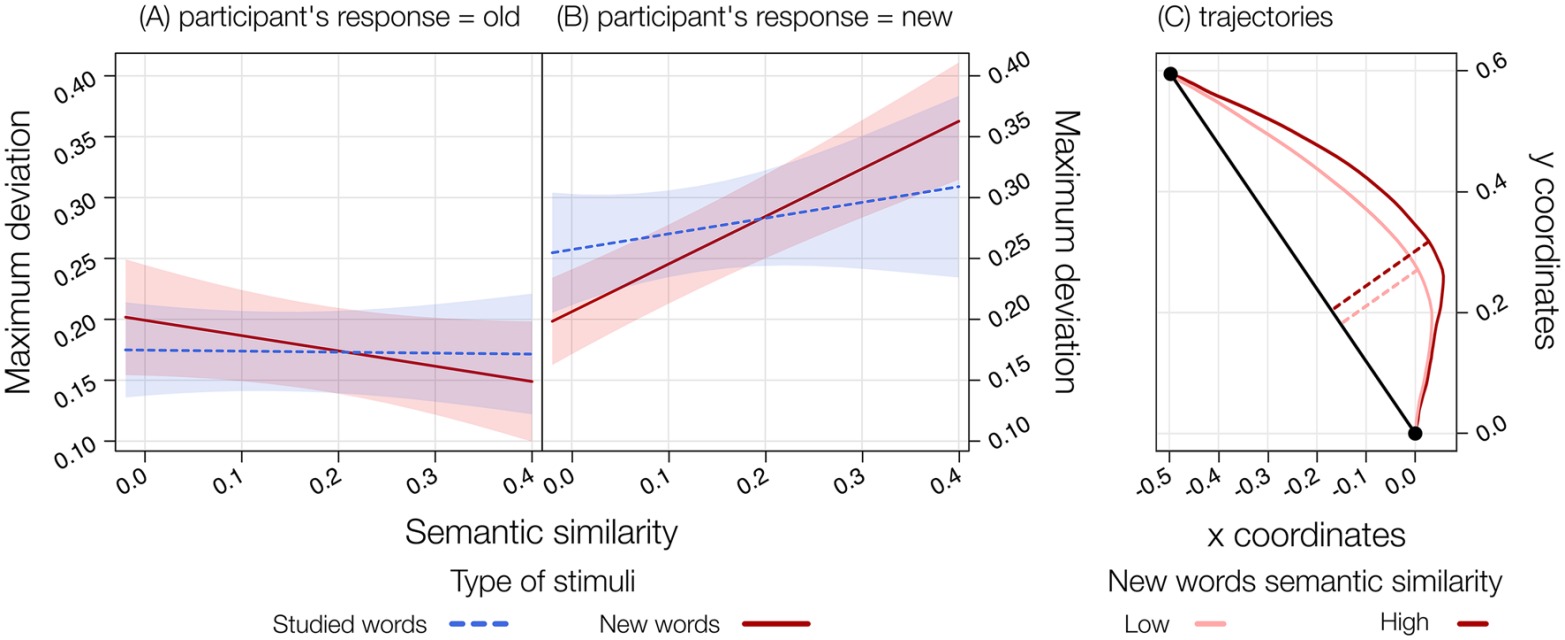
Results from the LMM on MD including the interaction between SSim, type of stimuli divided across participant’s response = old (**A**) and participant’s response = new (**B**), showing the positive relationship between SSim and MD when participants correctly rejected new words. A pictorial representation of how the maximum deviation (in dashed lines) from the direct path (black straight line) varied across two hypothetical levels of SSim (i.e., low and high SSim; the two categories were created by selecting the half of words with lower SSim and the other half with the higher SSim) of new words when participants correctly rejected them (**C**)

In the LMM including MD as a dependent variable, SSim, type of stimuli and participant’s response as predictors, we found significant effects of SSim, *F*(1, 6040) = 4.91, *p* = 0.02, of participant’s response, *F*(1, 6047) = 10.11, *p* = 0.001, of the interaction SSim by participant’s response, *F*(1, 6044) = 13.97, *p* < 0.001, of the interaction type of stimuli by participant’s response, *F*(1, 6045) = 7.22, *p* = 0.007, and, critically, of the three-way interaction SSim by type of stimuli by participant’s response, *F*(1, 6043) = 4.71, *p* = 0.02, *Pseudo-R*^2^ (total) = 0.27 (Fig. 5A and B).

The significant three-way interaction indicates that, when participants correctly rejected new words (i.e., participant’s response = new), SSim significantly predicted MD, *t*(6041) = 6.36, *p* < 0.001, *b* = 0.39. In particular, the higher the SSim for new words, the higher participants’ degree of indecision when correctly rejecting it (Fig. 4C). No effect of SSim was found when participants reported as old both new, *t*(6041) = − 1.54, *p* = 0.12, *b* = − 0.12, and studied words, *t*(6040) = − 0.11, *p* = 0.90, *b* = − 0.008, or when rejecting studied words, *t*(6043) = 1.05, *p* = 0.29, *b* = 0.12. Observed power = 99.70%.

#### Sample entropy

**Fig. 6:**
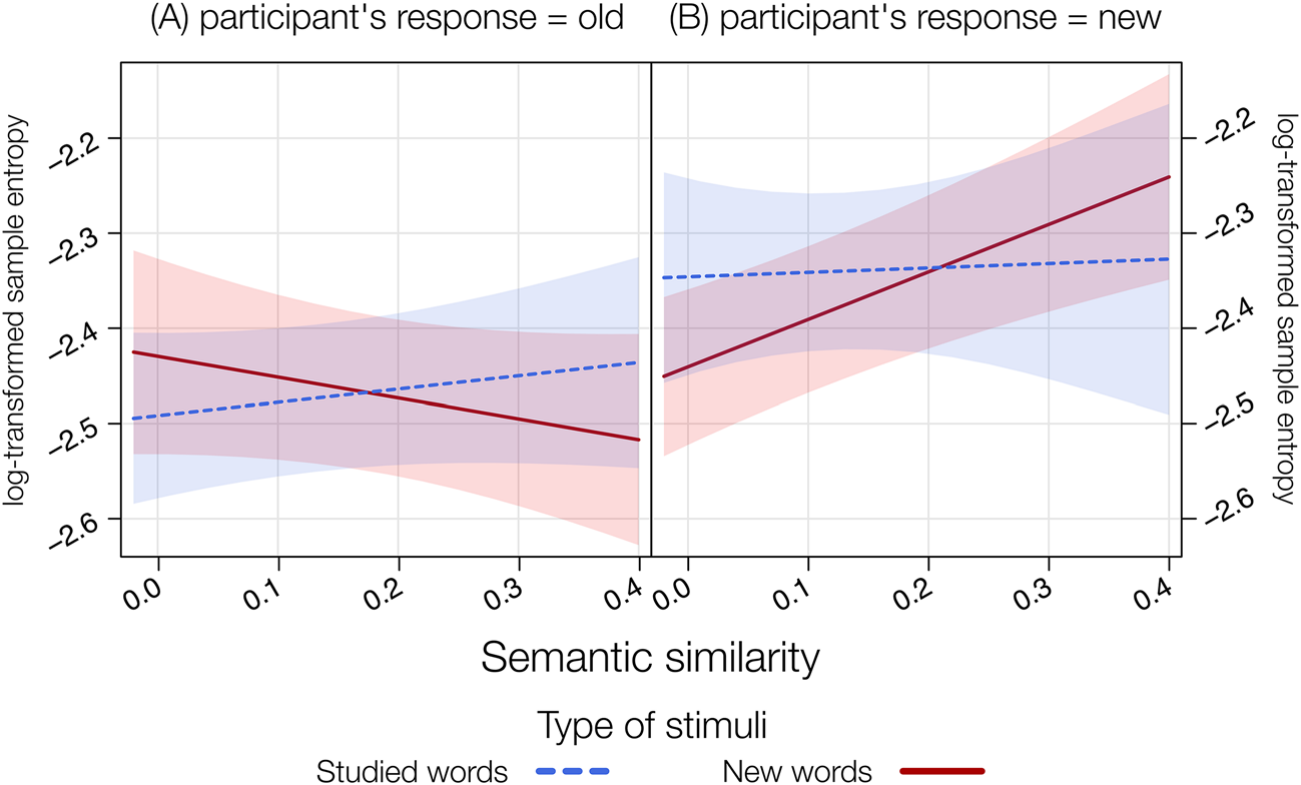
Results from the LMM on log-transformed entropy including the interaction between SSim, type of stimuli divided across participant’s response = old (**A**) and participant’s response = new (**B**), showing the positive relationship between SSim and log-transformed entropy when participants correctly rejected new words

In the LMM including sample entropy as a dependent variable, SSim, type of stimuli and participant’s response as predictors, we found significant effects of participant’s response, *F*(1, 6083) = 4.98, *p* = 0.02, on the interaction type of stimuli by participant’s response, *F*(1, 6082) = 6.80, *p* = 0.009 and, critically, of the three-way interaction SSim by type of stimuli by participant’s response, *F*(1, 6080) = 4.68, *p* = 0.03, *Pseudo-R*^2^ (total) = 0.27 (Fig. 6).

The significant three-way interaction indicates that, when participants correctly rejected new words (i.e., participant’s response = new), SSim significantly predicted sample entropy, *t*(6079) = 6.79, *p* < 0.001, *b* = 0.50. In particular, the higher the SSim between the new words, the higher the participants’ degree of indecision when correctly rejecting it. No effect of SSim was found when participants reported as old both new, *t*(6079) = − 1.26, *p* = 0.21, *b* = − 0.22, and studied words, *t*(6078) = 0.91, *p* = 0.36, *b* = 0.14, or when rejecting studied words, *t*(6080) = 0.18, *p* = 0.86, *b* = 0.05. Observed power = 79.30%.

#### MDtime

**Fig. 7:**
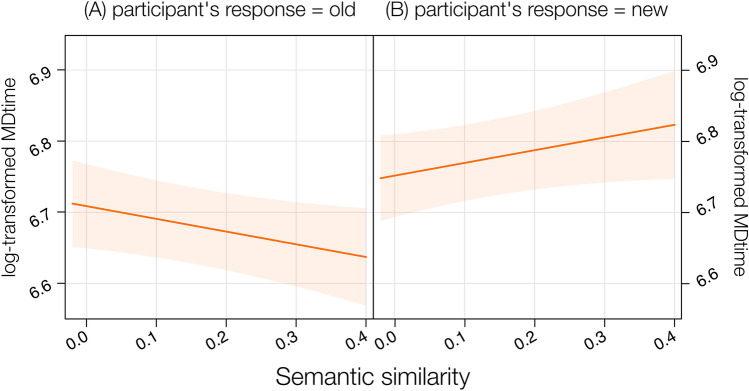
Results from the LMM on log-transformed MDtime including the interaction between SSim, type of stimuli divided across participant’s response = old (**A**) and participant’s response = new (**B**), showing the negative relationship between SSim and MDtime when participants recognized as old both types of stimuli (left) and the positive relationship between SSim and MDtime when participants rejected both types of stimuli (right)

In the LMM including MDtime as a dependent variable, SSim, type of stimuli and participant’s response as predictors, we found significant effects of participant’s response, *F*(1, 4783) = 6.28, *p* = 0.01, on the interaction type of stimuli by participant’s response, *F*(1, 4783) = 8.05, *p* = 0.004 and of the interaction SSim by participant’s response, *F*(1, 4899) = 10.94, *p* < 0.001, *Pseudo-R*^*2*^ (total) = 0.39 (Fig. 7). On the contrary, the three-way interaction SSim by type of stimuli by participant’s response was not significant, *F*(1, 4912) = 1.29, *p* = 0.25.

The significant interaction SSim by participant’s response indicates that, regardless of the type of stimuli, the higher the SSim, the faster participants were in deciding that a word was old, *t*(280) = − 2.10, *p* = 0.04, *b* = − 0.26; conversely, the higher the SSim, the slower participants were in rejecting a word (i.e., identifying it as new), *t*(153) = 2.22, *p* = 0.03, *b* = 0.22. Observed power = 96.00%.

## Discussion

In the present study, we explored the decisional stages subserving recognition memory in the DRM task taking advantage of mouse-tracking and of distributional semantic models. Participants were asked to memorize several lists of words in a classic DRM task and then to recognize them among new words using their mouse. The decision-making processes were indexed through different variables computed from mouse trajectories and predicted through item-level semantic metrics extracted from distributional semantic models (for a complete discussion see: Gatti et al., 2022). Overall, our findings indicate that mouse trajectories are affected by the semantic similarity between each word in the recognition phase and the previously studied words. Specifically, our findings indicate that mouse trajectories are affected by the semantic similarity between each word in the recognition phase and the previously studied words. That is, the higher the semantic similarity, the higher the conflict driving the choice and the irregularity in the trajectory (respectively, measured with the maximum deviation from the direct path and with sample entropy) when correctly rejecting new words. Conversely, on the temporal evolution of the decision, our findings indicate that semantic similarity predicts complex temporal measures indexing the online decision processes subserving task performance. More specifically, we found that regardless of the type of stimuli (old or new), when responding that a word was “old”, the higher the semantic similarity, the earlier the stage at which the decision was achieved; on the contrary, when rejecting a word (i.e., when responding that a word was “new”), the higher the semantic similarity, the later the stage at which the decision was achieved.

These findings well complement the key assumptions of the two main theories accounting for false memory in the DRM task, namely activation-monitoring framework and fuzzy-trace theory. Indeed, both theories trace back the origin of false recognitions to associative/semantic mechanisms, with adequate episodic and source memory processes that would counter them and enhance the occurrence of veridical recognition (Brainerd et al., 2002; Gallo & Roediger, 2002; Reyna & Brainerd, 1995; Roediger et al., 2001; and for individual differences evidence see: Gatti et al., 2021a). In interpreting our findings considering these theories, we first note that the dependent variable used to measure the conflict in the decision (maximum deviation from the direct path) quantifies it as an implicit measure of the attractiveness of the unselected option (Freeman et al., 2010). Thus, the higher the attractiveness of the unselected option, the higher the maximum deviation value because mouse trajectories would be associated with a larger curvature. Accordingly, our results show that even when participants correctly rejected new words (i.e., by selecting the “new” button), the “old” button exerted high attractivity. Notably, the level of attractiveness varied as a function of the semantic similarity, with a greater level of conflict for more semantically similar new words. Hence, while previous studies have shown that semantic memory is involved in the production of false memories (Gatti et al., 2022; see also: Montefinese et al., 2015), here we demonstrate that in the DRM task semantic processes participate also when correctly rejecting new words (i.e., the false memory items). Such an interpretation holds as well for the degree of irregularity and unpredictability of mouse movements, as the evolution of the choice was similarly affected by semantic similarity. That is, when correctly rejecting new words, movement irregularity was higher for more semantically similar new words.

The increased conflict in the rejection of new words as a function of their semantic similarity can be interpreted in terms of the alleged conflict emerging when participants are requested to judge if a new word was actually studied or not. In particular, activation-monitoring framework assumes that the critical lure is associatively hyperactivated in the encoding phase and then, in the recognition phase, such hyperactivation would be responsible for the false recognition (Gallo & Roediger, 2002; Roediger et al., 2001). On the other hand, fuzzy-trace theory assumes that while studying the word lists the participants would encode two memory traces: a semantic one, linked to the semantic content of each list; and an episodic one, linked to the contextual and perceptual features. In the recognition of a new word, these two traces would therefore counter each other (i.e., the semantic trace would increase the likelihood of false recognition, while the episodic trace would operate in the opposite direction), while for studied words they would both boost veridical recognition (Brainerd et al., 2002; Reyna & Brainerd, 1995). The enhanced conflict and uncertainty in participants’ rejection of new words with increasing semantic similarity observed here directly documents the online decision processes underpinning false memories, thus supporting both the activation-monitoring framework and fuzzy-trace theory. That is, for the new words in which the episodic trace is lacking, the conflict and the uncertainty would increase with increased semantic similarity of the new word. Specifically, our study shows that using mouse-tracking, it is possible to extract several behavioral metrics indexing the conflict and uncertainty underlying human performance in the DRM task. Additionally, the fact that, across old responses, new and studied words share similar conflict and timing indexes as extracted from mouse trajectories can be considered as the implicit counterpart of the seminal evidence that confidence ratings for both falsely recognized critical lures and correctly recognized studied words show similar levels (e.g., Roediger & McDermott, 1995).

Previous studies investigating associative and semantic involvement in the DRM task have shown that backward associative strength is a major predictor of false memories (Roediger et al., 2001; but see also: Brainerd et al., 2020), that multiple semantic sub-components underlie false memories (Cann et al., 2011) and that memory performance follows a continuous semantic gradient (Gatti et al., 2022). This last finding was replicated in this study, by observing that for new words, the higher the semantic similarity value, the higher the occurrence of false memories. Critically, here we extended this evidence by showing that the semantic similarity between the words presented in the recognition phase and those previously studied affects not only the explicit memory judgements (i.e., “yes” and “no” recognition responses), but also more implicit measures extracted from participants’ motor outputs. These findings support previous evidence, in that they suggest that, while memorizing the words, participants would implicitly and automatically activate the semantic trace for each list and would then use this trace during the recognition task when judging new words (Gatti et al., 2022). This effect has been explained by arguing that, since during the encoding task participants were shown the lists of words without any clues that clustered the words, the sequential presentation of each word within each list would have incrementally activated a meaning cluster composed of the same words in semantic memory (Gatti et al., 2022). Here, extending upon these findings, we further show that semantic similarity between studied and new words affects decision-making in memory retrieval: the more the cluster of words composing each list is semantically close to the new word, the higher the participants’ conflict and uncertainty when correctly rejecting new words. This indicates, therefore, that the structure of semantic memory and the activation of specific clusters of words affect memory retrieval, with the degree of overlap between the vectors representing new and studied words accounting for the level of conflict in the decision-making process.

For the main timing measure (i.e., the time at which the mouse trajectory finally deviates), we found that the semantic similarity affected participants’ performance in both veridical and false memories. In particular, the higher the semantic similarity, the faster participants were in deciding that a word was old, and the opposite for “new” judgments. Hence, the time at which the decision occurred was influenced in opposite directions by semantic similarity, indicating that this variable overall impacted the temporal dynamics subserving task performance. This dissociation may suggest that semantic memory involvement in the DRM task could affect differently temporal and spatial measures of decision making, thus dissociating the time needed to decide if a word was old or new from deeper decision-making components, such as the conflict and indecision underlying memory judgements. As maintained by the activation-monitoring framework and fuzzy-trace theory, different cognitive processes (i.e., associative/semantic and episodic) come simultaneously into play in the DRM task, generating in turn different outcomes (Brainerd et al., 2002; Gallo & Roediger, 2002; Reyna & Brainerd, 1995; Roediger et al., 2001). Specifically, the conflict observable in the spatial measures that can be traced back to the interplay between associative/semantic and episodic traces was not observed in the main timing measure, suggesting that two main decision components (i.e., spatial and temporal) are active in parallel during memory retrieval in the DRM task. This dissociation can be explained through dynamical decision-making frameworks arguing that several explicit and implicit processes simultaneously compete when making a decision (Freeman & Ambady, 2011; Melnikoff & Bargh, 2018).

Our findings have relevant implications from both theoretical and methodological points of view. On a theoretical level, our results clarify semantic memory involvement in a complex memory task such as the DRM when participants’ performance is measured through fine-grained hand movements. In particular, here we provide evidence for possible differential involvement of semantic memory across time, conflict and uncertainty of participants’ decisions. Additionally, by successfully predicting mouse-tracking measures using a semantic predictor extracted from a distributional semantic model, we provide further support to the idea that these models are extremely efficient in capturing the structure of human semantic memory (Günther et al., 2019). Indeed, while previous studies have predicted participants’ performance using distributional semantic models across a wide range of semantic tasks, such as multiple-choice tests (Bullinaria & Levy, 2007), word categorization (Baroni & Lenci, 2010), word relatedness ratings (Bruni et al., 2014), word naming and lexical decision (Marelli & Amenta, 2018), semantic priming (Günther et al., 2016), recognition memory (Gatti et al., 2021c, 2022), as well as using mouse-tracking (Gatti et al., 2021b), our study is the first to report its effect also on mouse-tracking variables in a complex memory task such as the DRM. Furthermore, on a methodological level, we show that by pairing distributional semantic models with mouse-tracking it is possible to investigate deep decision-making features of human behavior, thus opening new avenues for probing the detailed processes subserving human memory, with this method being promising also for specific manipulations in the DRM task, such as when testing the effect of warning on false memories. The methods used here may be as well particularly suited for better understanding individual differences in false memories. This is indeed a topic of intense research (e.g., Ball et al., 2021; Leding, 2011, 2012; Nichols & Loftus, 2019; Unsworth & Brewer, 2010; Watson et al., 2005), and mouse-tracking could provide additional insights regarding implicit and semantic processes involved in false remembering. For instance, recent evidence has shown that individuals with higher source-monitoring abilities are better at recalling contextual information from encoding to correctly reject lures (Ball et al., 2021). Future studies could thus address to what extent individuals with better source-monitoring abilities would also manifest decreased indecision and conflict when rejecting a critical lure as measured with mouse-tracking.

In conclusion, using distributional semantic models combined with mouse-tracking, we document the decision-making semantic processes underpinning false memories. Our findings are consistent with previous theories on participants’ behavior in the DRM task and provide novel insights into the impact of semantic memory on different decision-making components.

## Supplementary Information

Below is the link to the electronic supplementary material.

Supplementary file1 (XLS 1892 KB)

## Footnotes

1 The relative cosine values used in this Experiment can be reproduced through the freely available user-friendly interface at: http://meshugga.ugent.be/snaut-italian/. Note that the produced distance estimates were subtracted from 1 to obtain a proximity scale.

2 Note that also when computing the correlations separately on data for new vs. studied items or old vs. new responses we found similar *r* values.
